# Supplementary figures and images for: Temporal changes in the structure of a plant-frugivore network are influenced by bird migration and fruit availability
Source: PeerJ. 2016 Jun 8;4:e2048. doi: 10.7717/peerj.2048 (PMC4906665; doi:10.7717/peerj.2048)

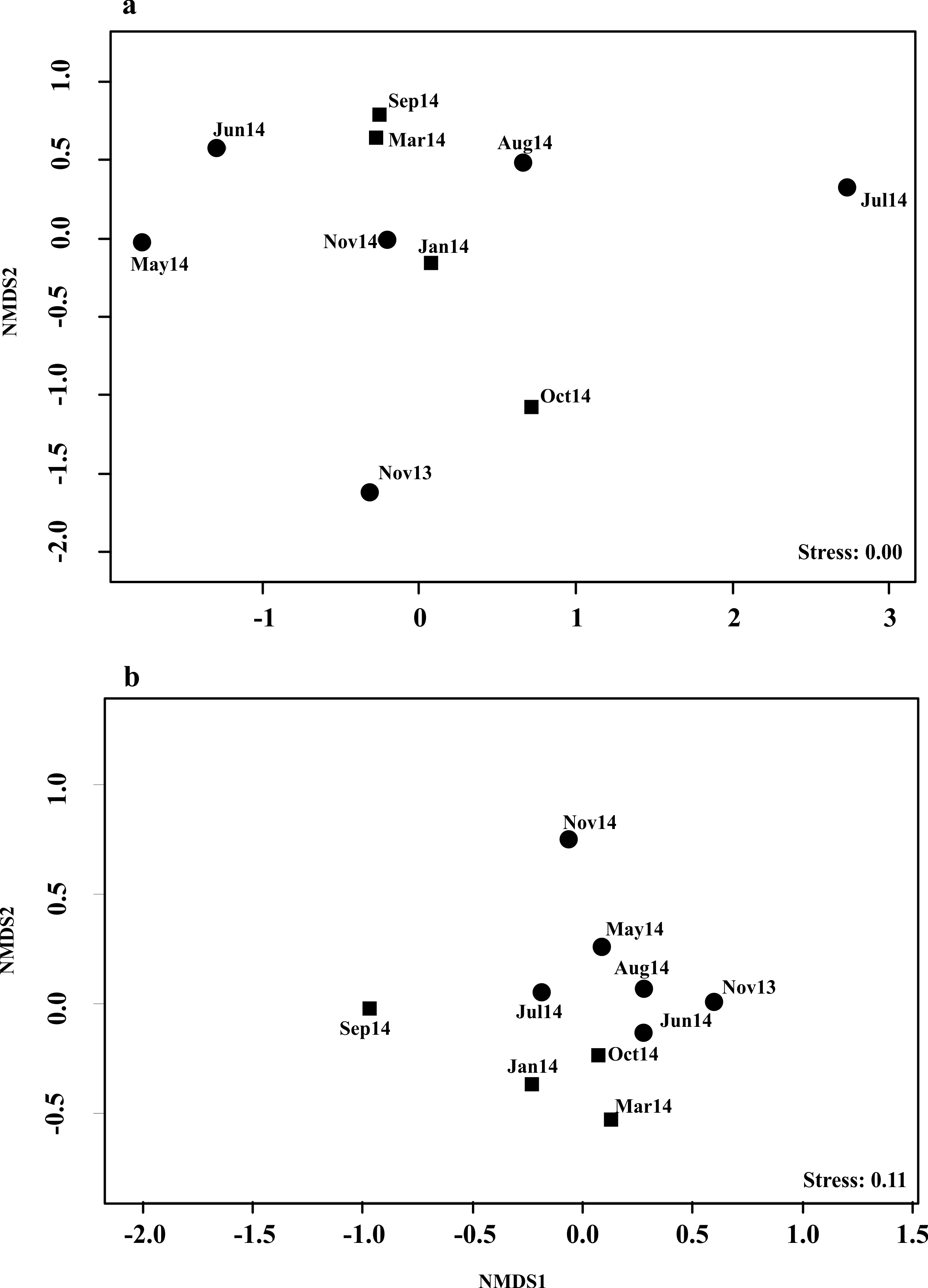

Supplement: Figure S1 — Non-metric multidimensional scaling (NMDS) ordinations for the composition of frugivorous bird species at the network core (a) and the network periphery (b). To carry out the analysis and ordination samplings were a priori grouped into two categories: i) high proportion of migratory bird species (circles): Jan14, Mar14, Sep14 and Oct14; and, low proportion of migratory birds species (square): Nov13, May14, Jun14, Jul14, Aug 14 and Nov14. [file peerj-04-2048-s001.png]
