# Supplementary material for: Temporal changes in the structure of a plant-frugivore network are influenced by bird migration and fruit availability
Source: PeerJ. 2016 Jun 8;4:e2048. doi: 10.7717/peerj.2048 (PMC4906665; doi:10.7717/peerj.2048)
Supplement: Table S1 — Species list of frugivorous birds and plants they interact with. ID contains the codes depicted at the graphs. Birds were classified according to their migratory status (resident and migrant). [file peerj-04-2048-s002.docx]

| **Supplemental Information 1. List of frugivores and plants.** | | | | | | | | | | |  |  |  |  |  |  |  |  |  |  |  |  |  |  |  |
| --- | --- | --- | --- | --- | --- | --- | --- | --- | --- | --- | --- | --- | --- | --- | --- | --- | --- | --- | --- | --- | --- | --- | --- | --- | --- |
| **Table S1.** List of species of fruit-eating birds and plants with which they interact. Birds were classified according to their migration status (resident or migratory). Bird species of each network that belong to generalist core network (black) and the periphery (gray). Plant species of each network (gray). | | | | | | | | | | | | | | | | | | | | | | | | | |
|  | **Frugivores** |  | **Networks** | | | | | | | | | |  |  | **Plants** | **Networks** | | | | | | | | | |
| **ID** | **Species** | **Status** | **1** | **2** | **3** | **4** | **5** | **6** | **7** | **8** | **9** | **10** |  | **ID** | **Species** | **1** | **2** | **3** | **4** | **5** | **6** | **7** | **8** | **9** | **10** |
| B1 | *Eupsittula nana* | Resident |  |  |  |  |  |  |  |  |  |  |  | P1 | *Achatocarpus nigricans* Triana |  |  |  |  |  |  |  |  |  |  |
| B2 | *Campylorhynchus rufinucha* | Resident |  |  |  |  |  |  |  |  |  |  |  | P2 | *Bursera fagaroides* (Kunth) Engl. |  |  |  |  |  |  |  |  |  |  |
| B3 | *Dives dives* | Resident |  |  |  |  |  |  |  |  |  |  |  | P3 | *Bursera simaruba* (L.) Sarg. |  |  |  |  |  |  |  |  |  |  |
| B4 | *Dumetella carolinensis* | Migratory |  |  |  |  |  |  |  |  |  |  |  | P4 | *Casearia corymbosa* Kunth |  |  |  |  |  |  |  |  |  |  |
| B5 | *Empidonax virescens* | Migratory |  |  |  |  |  |  |  |  |  |  |  | P5 | *Chiococca alba* (L.) Hitchc. |  |  |  |  |  |  |  |  |  |  |
| B6 | *Hylocichla mustelina* | Migratory |  |  |  |  |  |  |  |  |  |  |  | P6 | *Coccoloba barbadensis* Jacq. |  |  |  |  |  |  |  |  |  |  |
| B7 | *Icteria virens* | Migratory |  |  |  |  |  |  |  |  |  |  |  | P7 | *Cordia dentata* Poir. |  |  |  |  |  |  |  |  |  |  |
| B8 | *Icterus galbula* | Migratory |  |  |  |  |  |  |  |  |  |  |  | P8 | *Crateva tapia* L. |  |  |  |  |  |  |  |  |  |  |
| B9 | *Icterus graduacauda* | Resident |  |  |  |  |  |  |  |  |  |  |  | P9 | *Chrysobalanus icaco* L. |  |  |  |  |  |  |  |  |  |  |
| B10 | *Icterus gularis* | Resident |  |  |  |  |  |  |  |  |  |  |  | P10 | *Crossopetalum uragoga* (Jacq.) Kuntze |  |  |  |  |  |  |  |  |  |  |
| B11 | *Icterus spurius* | Migratory |  |  |  |  |  |  |  |  |  |  |  | P11 | *Cupania dentata* Moc. & Sessé ex DC. |  |  |  |  |  |  |  |  |  |  |
| B12 | *Leptotila verreauxi* | Resident |  |  |  |  |  |  |  |  |  |  |  | P12 | *Dendropanax arboreus* (L.) Decne. & Planch. | |  |  |  |  |  |  |  |  |  |
| B13 | *Megarynchus pitangua* | Resident |  |  |  |  |  |  |  |  |  |  |  | P13 | *Diospyros verae-crucis* (Standl.) Standl. |  |  |  |  |  |  |  |  |  |  |
| B14 | *Melanerpes aurifrons* | Resident |  |  |  |  |  |  |  |  |  |  |  | P14 | *Ehretia tinifolia* L. |  |  |  |  |  |  |  |  |  |  |
| B15 | *Myiarchus crinitus* | Migratory |  |  |  |  |  |  |  |  |  |  |  | P15 | *Ficus cotinifolia* Kunth |  |  |  |  |  |  |  |  |  |  |
| B16 | *Myiarchus tuberculifer* | Resident |  |  |  |  |  |  |  |  |  |  |  | P16 | *Ficus insipida* Willd. |  |  |  |  |  |  |  |  |  |  |
| B17 | *Myiarchus tyrannulus* | Resident |  |  |  |  |  |  |  |  |  |  |  | P17 | *Ficus* sp |  |  |  |  |  |  |  |  |  |  |
| B18 | *Myiodynastes luteiventris* | Migratory |  |  |  |  |  |  |  |  |  |  |  | P18 | *Karwinskia humboldtiana* (Schult.) Zucc. |  |  |  |  |  |  |  |  |  |  |
| B19 | *Myiozetetes similis* | Resident |  |  |  |  |  |  |  |  |  |  |  | P19 | *Maclura tinctoria* (L.) D.Don ex Steud. |  |  |  |  |  |  |  |  |  |  |
| B20 | *Ortalis vetula* | Resident |  |  |  |  |  |  |  |  |  |  |  | P20 | *Nectandra salicifolia* (Kunth) Nees |  |  |  |  |  |  |  |  |  |  |
| B21 | *Pachyramphus aglaiae* | Resident |  |  |  |  |  |  |  |  |  |  |  | P21 | *Opuntia stricta* (Haw.) Haw. |  |  |  |  |  |  |  |  |  |  |
| B22 | *Passerina caerulea* | Migratory |  |  |  |  |  |  |  |  |  |  |  | P22 | *Paullinia tomentosa* Jacq. |  |  |  |  |  |  |  |  |  |  |
| B23 | *Patagioenas flavirostris* | Resident |  |  |  |  |  |  |  |  |  |  |  | P23 | *Psittacanthus calyculatus* (DC.) G.Don |  |  |  |  |  |  |  |  |  |  |
| B24 | *Pheucticus ludovicianus* | Migratory |  |  |  |  |  |  |  |  |  |  |  | P24 | *Psychotria erythrocarpa* Schltdl. |  |  |  |  |  |  |  |  |  |  |
| B25 | *Piaya cayana* | Resident |  |  |  |  |  |  |  |  |  |  |  | P25 | *Rourea glabra* Kunth |  |  |  |  |  |  |  |  |  |  |
| B26 | *Piranga rubra* | Migratory |  |  |  |  |  |  |  |  |  |  |  | P26 | *Smilax aristolochiifolia* Mill. |  |  |  |  |  |  |  |  |  |  |
| B27 | *Pitangus sulphuratus* | Resident |  |  |  |  |  |  |  |  |  |  |  | P27 | *Solanum diphyllum* L. |  |  |  |  |  |  |  |  |  |  |
| B28 | *Polioptila caerulea* | Resident |  |  |  |  |  |  |  |  |  |  |  | P28 | Sp1 |  |  |  |  |  |  |  |  |  |  |
| B29 | *Psarocolius montezuma* | Resident |  |  |  |  |  |  |  |  |  |  |  | P29 | Sp2 |  |  |  |  |  |  |  |  |  |  |
| B30 | *Psilorhinus morio* | Resident |  |  |  |  |  |  |  |  |  |  |  | P30 | Sp3 |  |  |  |  |  |  |  |  |  |  |
| B31 | *Quiscalus mexicanus* | Resident |  |  |  |  |  |  |  |  |  |  |  | P31 | Sp4 |  |  |  |  |  |  |  |  |  |  |
| B32 | *Thraupis abbas* | Resident |  |  |  |  |  |  |  |  |  |  |  | P32 | Sp5 |  |  |  |  |  |  |  |  |  |  |
| B33 | *Thraupis episcopus* | Resident |  |  |  |  |  |  |  |  |  |  |  | P33 | Sp6 |  |  |  |  |  |  |  |  |  |  |
| B34 | *Tityra semifasciata* | Resident |  |  |  |  |  |  |  |  |  |  |  | P34 | Sp7 |  |  |  |  |  |  |  |  |  |  |
| B35 | *Trogon melanocephalus* | Resident |  |  |  |  |  |  |  |  |  |  |  | P35 | Sp8 |  |  |  |  |  |  |  |  |  |  |
| B36 | *Turdus grayi* | Resident |  |  |  |  |  |  |  |  |  |  |  | P36 | Sp9 |  |  |  |  |  |  |  |  |  |  |
| B37 | *Tyrannus forficatus* | Migratory |  |  |  |  |  |  |  |  |  |  |  | P37 | Sp10 |  |  |  |  |  |  |  |  |  |  |
| B38 | *Tyrannus melancholicus* | Resident |  |  |  |  |  |  |  |  |  |  |  | P38 | Sp11 |  |  |  |  |  |  |  |  |  |  |
| B39 | *Tyrannus tyrannus* | Migratory |  |  |  |  |  |  |  |  |  |  |  | P39 | Sp12 |  |  |  |  |  |  |  |  |  |  |
| B40 | *Vireo griseus* | Migratory |  |  |  |  |  |  |  |  |  |  |  | P40 | Sp13 |  |  |  |  |  |  |  |  |  |  |
| B41 | *Vireo philadelphicus* | Migratory |  |  |  |  |  |  |  |  |  |  |  | P41 | *Stemmadenia galeottiana* (A.Rich.) Miers |  |  |  |  |  |  |  |  |  |  |
| B42 | *Vireo solitarius* | Migratory |  |  |  |  |  |  |  |  |  |  |  | P42 | *Trichilia hirta* L. |  |  |  |  |  |  |  |  |  |  |
|  |  |  |  |  |  |  |  |  |  |  |  |  |  | P43 | *Vitis bourgaeana* Planch. |  |  |  |  |  |  |  |  |  |  |
|  |  |  |  |  |  |  |  |  |  |  |  |  |  | P44 | *Xylosma panamensis* Turcz. |  |  |  |  |  |  |  |  |  |  |
