# Supplementary material for: Temporal changes in the structure of a plant-frugivore network are influenced by bird migration and fruit availability
Source: PeerJ. 2016 Jun 8;4:e2048. doi: 10.7717/peerj.2048 (PMC4906665; doi:10.7717/peerj.2048)
Supplement: Table S2 — Relation of fruit availability (fruit richness and abundance) and bird migration with parameters of bird-plant networks at CICOLMA. Significant relations were in bold (P ≤ 0.05). [file peerj-04-2048-s003.docx]

| **Supplemental Information 2. Relation between fruit availability and proportion of migratory birds.** | | | | | |
| --- | --- | --- | --- | --- | --- |
| **Table S2.** Relationships between explanatory variables (fruit richness, fruit abundance and proportion of migratory bird species) and networks parameters as response variables, at CICOLMA. Significant relationships are shown with bold values. | | | | | |
|  | Parameter | Estimate | (± ES) | *z/t* value | *P* |
| **a.** | **Network size (NS)** |  |  |  |  |
|  | Proportion of migratory birds | **0.032** | **0.013** | **2.460** | **0.013** |
|  | Richness | **0.147** | **0.052** | **2.829** | **0.004** |
|  | Proportion of migratory birds * Richness | **-0.004** | **0.001** | **-2.671** | **0.007** |
|  | Abundance | -0.244 | 0.231 | -1.057 | 0.321 |
| **b.** | **Connectance (C)** |  |  |  |  |
|  | Proportion of migratory birds | **-0.027** | **0.011** | **-2.389** | **0.050** |
|  | Richness | **-0.150** | **0.047** | **-3.165** | **0.019** |
|  | Proportion of migratory birds * Richness | 0.002 | 0.001 | 2.015 | 0.090 |
|  | Abundance | **0.689** | **0.134** | **5.121** | **0.001** |
| **c.** | **Nestedness (NODF)** |  |  |  |  |
|  | Proportion of migratory birds | **-0.024** | **0.01** | **-2.266** | **0.023** |
|  | Richness | **-0.123** | **0.043** | **-2.815** | **0.004** |
|  | Proportion of migratory birds * Richness | 0.002 | 0.001 | 1.940 | 0.052 |
|  | Abundance | **0.577** | **0.139** | **4.157** | **0.003** |
| **d.** | **Specialization (H_2_)** |  |  |  |  |
|  | Proportion of migratory birds | 0.027 | 0.016 | 1.656 | 0.148 |
|  | Richness | 0.064 | 0.068 | 0.949 | 0.379 |
|  | Proportion of migratory birds * Richness | -0.002 | 0.002 | -1.379 | 0.217 |
|  | Abundance | -0.317 | 0.187 | -1.691 | 0.129 |
| **e.** | **Interaction strength assymmetry (ISA)** |  |  |  |  |
|  | Proportion of migratory birds | **0.042** | **0.015** | **2.688** | **0.036** |
|  | Richness | -0.090 | 0.067 | -1.332 | 0.231 |
|  | Proportion of migratory birds * Richness | -0.004 | 0.002 | -1.740 | 0.132 |
|  | Abundance | -0.007 | 0.422 | -0.018 | 0.986 |
| **f.** | **Niche overlap (birds)** |  |  |  |  |
|  | Proportion of migratory birds | -0.001 | 0.026 | -0.045 | 0.966 |
|  | Richness | -0.243 | 0.123 | -1.965 | 0.097 |
|  | Proportion of migratory birds * Richness | 0.002 | 0.003 | 0.669 | 0.529 |
|  | Abundance | 0.415 | 0.426 | 0.974 | 0.359 |
|  |  |  |  |  |  |
